# A double blind, placebo-controlled randomized comparative study on the efficacy of phytosterol-enriched and conventional saw palmetto oil in mitigating benign prostate hyperplasia and androgen deficiency

**DOI:** 10.1186/s12894-020-00648-9

**Published:** 2020-07-03

**Authors:** H. V. Sudeep, Jestin V. Thomas, K. Shyamprasad

**Affiliations:** 1 R&D Center for Excellence, Vidya Herbs Pvt. Ltd, #14A, Jigani I phase, Bangalore, Karnataka 560 105 India; 2 Leads Clinical Research and Bio services Private Ltd., Bangalore, India

**Keywords:** Saw palmetto oil, β-Sitosterol, Benign prostate hyperplasia, Androgen deficiency, Safety

## Abstract

**Background:**

The present clinical trial was conducted to evaluate the efficacy and tolerability of a standardized saw palmetto oil containing 3% β-sitosterol in the treatment of benign prostate hyperplasia (BPH) and androgen deficiency.

**Methods:**

Subjects aged 40–65 years with symptomatic BPH were randomized to 12-week double-blind treatment with 500 mg doses of β-sitosterol enriched saw palmetto oil, conventional saw palmetto oil and placebo orally in the form of capsules (*n* = 33 in each group). BPH severity was determined using the International Prostate Symptom Score (IPSS), uroflowmetry, serum measurement of prostate specific antigen (PSA), testosterone and 5α-reductase. During the trial, the androgen deficiency was evaluated using Aging Male Symptoms (AMS) scale, the Androgen Deficiency in the Aging Male (ADAM) questionnaire, serum levels of free testosterone.

**Results:**

Subjects treated with β-sitosterol enriched saw palmetto oil showed significant decrease in IPSS, AMS and ADAM scores along with reduced postvoiding residual volume (*p* < 0.001), PSA (*p* < 0.01) and 5α-reductase from baseline to end of 12-week treatment as compared to placebo. There was also a significant increment in the maximum and average urine flow rate (*p* < 0.001), and serum free testosterone level of subjects treated with enriched saw palmetto oil as compared to placebo.

**Conclusion:**

This study demonstrates the efficacy of β-sitosterol enriched saw palmetto oil superior to conventional oil thus extending the scope of effective BPH and androgen deficiency treatment with improved quality of life through the intake of functional ingredients.

**Trial registration:**

CTRI/2018/12/016724 dated 19/12/2018 prospectively registered. URL: http://ctri.nic.in/Clinicaltrials/advsearch.php

## Background

Growing interest in the use of functional foods and dietary supplements for healthcare management has led to the availability of natural products in the market. However, development of nutraceuticals requires supportive evidence of efficacy and tolerability that must be clearly demonstrated with clinical trials [1]. *Serenoa repens* (saw palmetto) is one of the most studied plants for the treatment of lower urinary tract diseases (LUTS), especially mild to moderate prostatic diseases. Major bioactive principles attributing to the medicinal use of saw palmetto oil are the phytosterols and fatty acids. Explored mechanisms of action for saw palmetto oil includes inhibition of 5α-reductase, cyclooxygenase (COX) and 5-lipoxygenase (LOX). In addition, it exhibits antiproliferative effect on prostatic epithelial cells and antiestrogenic activity [2]. Thus, the candidature of saw palmetto preparations as alternative medicine to treat benign prostate hyperplasia has been the subject of clinical research.

Saw palmetto (SP) has been clinically tested either alone or in combination with other alpha-blockers and 5α-reductase inhibitors (5-ARIs). Guilianelli et al. demonstrated in a multicentre clinical trial that the use of SP extract for 6 months showed significant improvement in International Prostate Symptom (IPSS) score and uroflowmetry in subjects with BPH [3]. Sinescu et al. have reported the long-term efficacy of SP extract in improving the LUTS, urinary flow, erectile dysfunction and quality of life (QoL) [4]. In a recent double-blind, placebo-controlled study Ye et al. reported the improvements in LUTS and male sexual function followed by treatment with SP extract [5]. SP extract demonstrated equivalent efficacy when compared to treatments with other alpha-blockers and 5-ARIs [6–8].

Phytoconstituents such as sterols, fatty acids and vitamin E together contribute to the efficacy of SP extract in mitigating the BPH complications [9, 10]. Conventionally SP oil contains 0.2–0.3% β-sitosterol in addition to fatty acids. Here we have used a 3% β-sitosterol-containing SP oil for the clinical evaluation of therapeutic potential, alongside comparing with the conventional SP oil in a placebo-controlled trial. Previously we have demonstrated the improved efficacy of β-sitosterol-enriched SP oil as compared to the conventional oil in testosterone-induced BPH model rats [11]. Here we have provided the first ever clinical evidence on the improved efficacy of β-sitosterol-enriched SP extract superior to the conventional SP.

## Methods

### Investigational product

**Fig. 1:**
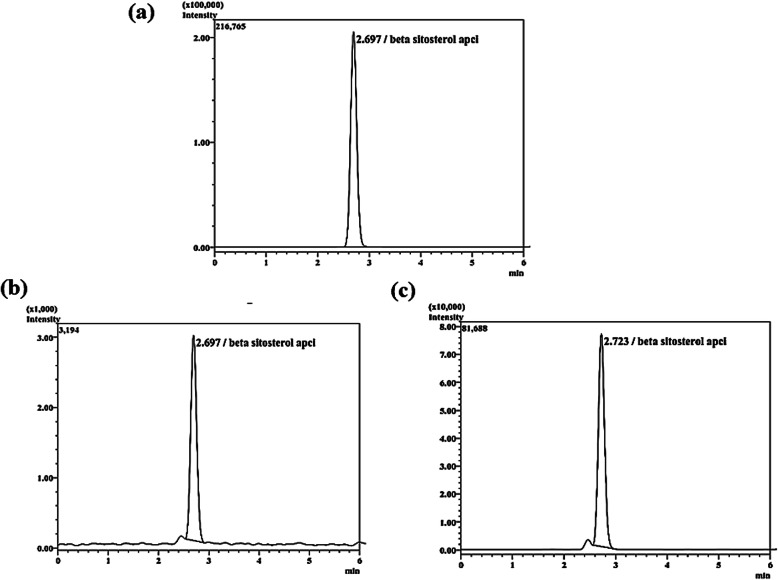
LCMS chromatograms of reference compound β-sitosterol (95%) (**a**), SPO (**b**) and VISPO (**c**)

The investigational product was a standardized saw palmetto extract containing 3% β-sitosterol (VISPO™). VISPO and the conventional saw palmetto oil (SPO, 0.2% β-sitosterol) were prepared in-house from the berries (Florida, USA) using the supercritical fluid extraction method. The higher percentage of β-sitosterol in VISPO was achieved by column chromatography. The β-sitosterol content was quantified using LCMS analysis (Fig. 1). The extracts were then formulated into powder form with maltodextrin as excipient and filled in capsules (500 mg in weight). Each capsule contained 200 mg of extract. Placebo capsules consisted of maltodextrin only.

### Ethics, consent and permissions

The Institutional Ethics Committee of Aman Hospital and Research Center, Vadodara, India approved the study protocol and informed consent form (AHRC/IEC/026/2018). This clinical study was registered in Clinical Trials Registry – India (CTRI/2018/12/016724 dated 19/12/2018).

### Subjects

Ninety-nine male subjects aged 40–65 years, having mild-to-moderate BPH symptoms were enrolled into this clinical trial. Before enrolment, subjects were explained the study protocol approved by the Institutional Ethics Committee, and informed consent and consent to publish were obtained. The subjects having IPSS score > 7, Aging Male Symptoms (AMS) score ≥ 27, and responding positively to question 1 or 7 or any other three questions of the Androgen Deficiency in the Aging Male (ADAM) questionnaire, were included in the trial. Subjects with neurogenic bladder dysfunction, prostatic cancer, prostatitis, haematuria diabetes or any other chronic illness were excluded from the study.

### Study design

This study was conducted in compliance with ICH-GCP (International Conference on Harmonization of Technical Requirements for Registration of Pharmaceuticals for Human Use –Good Clinical Practice) guidelines and Helsinki Declaration Standards. This clinical study adheres to the CONSORT guidelines.

The study was performed at Aman Hospital and Research Center, Vadodara, India. The trial was designed as a double-blind, placebo-controlled, single-centered study including 99 male subjects randomised to three treatment arms: VISPO, SPO and placebo, on 1:1:1 ratio (*n* = 33 in each group). Block randomization was implemented for groupwise allocation of subjects wherein the participants received a xx-digit randomization number. The investigator and subjects were blinded of the interventions. The study medications were dispensed by the unblinded pharmacist at the site.

The subjects received oral doses of 500 mg capsules twice daily (after breakfast and dinner) for 12 weeks. The study treatment details are provided in Table 1. Following baseline visit, during the intervention period there were two visits at study site every 6 weeks. The details of study procedures scheduled at each visit are provided in **supplementary file** **1**. The treatment compliance was monitored during the trial using subject diary.

**Table 1:** Details of study treatments

Group	Intervention (VISPO)	Comparator (SPO)	Placebo
Treatment	Saw palmetto oil (3% β-sitosterol)	Saw palmetto oil (0.2% β-sitosterol)	Maltodextrin
Dosage Form	Capsules	Capsules	Capsules
Composition	Capsule weight – 500 mg Saw palmetto oil – 200 mg Maltodextrin – 300 mg	Capsule weight – 500 mg Saw palmetto oil – 200 mg Maltodextrin – 300 mg	Capsule weight – 500 mg Maltodextrin – 500 mg
Batch No.	VH/SPO/F18716	VH/SPO/F18717	VH/MD/F18612
Route	Oral		
Dose	500 mg capsule two times a day; 400 mg/day of saw palmetto oil in active treatment groups.		
Dosing Regimen	Twice a day after food		
Treatment duration	84 days		

### Sample size

A sample size of 99 subjects (33 per group), was considered enough to detect a clinically important difference between groups with 80% power and a 5% level of significance. Considering an estimated potential dropout rate of 12%, the sample size was finalized as 99 (33 per group). For the sample size calculation IPSS and maximum urine flowrates were considered with a delta value of 2.35 (**Supplementary file** **2****)**.

### Study outcomes

The primary endpoints were determined as changes in IPSS, ADAM and AMS scores (subjective assessments). Objective assessment was based on post-void residual urine volume as measured by ultrasonography, and uroflowmetric determination of the maximum (Qmax) urine flowrate. The serum levels of prostate specific antigen (PSA), 5α-reductase and testosterone were measured at baseline and after 12 weeks of treatment using commercial kits. The general health status of subjects was assessed using the 12-tem Short Form Survey (SF-12) quality of life questionnaire. In SF-12, the questions are combined, scored, and weighted to create two scales that provide glimpses into mental and physical functioning and overall health-related-quality of life. Physical (PCS-12) and Mental Health Composite Scores (MCS-12) are computed using the scores of twelve questions and range from 0 to 100, where a zero score indicates the lowest level of health measured by the scales and 100 indicates the highest level of health. The questionnaires used in the study are detailed in **supplementary file** **3**.

The safety assessments included physical examination, vital signs, biochemical and hematological parameters (Aspartate aminotransferase (AST), alanine aminotransferase (ALT), serum creatinine, blood urea nitrogen (BUN) and complete blood count).

### Statistical analysis

The general characteristics of the study subjects are summarized as mean ± standard deviation (SD) for continuous data. ANOVA was performed when equal variances were assumed to test between groups. Kruskal-Wallis test was performed when equal variances are not assumed. Paired t test was performed to test variables within groups.

## Results

**Fig. 2:**
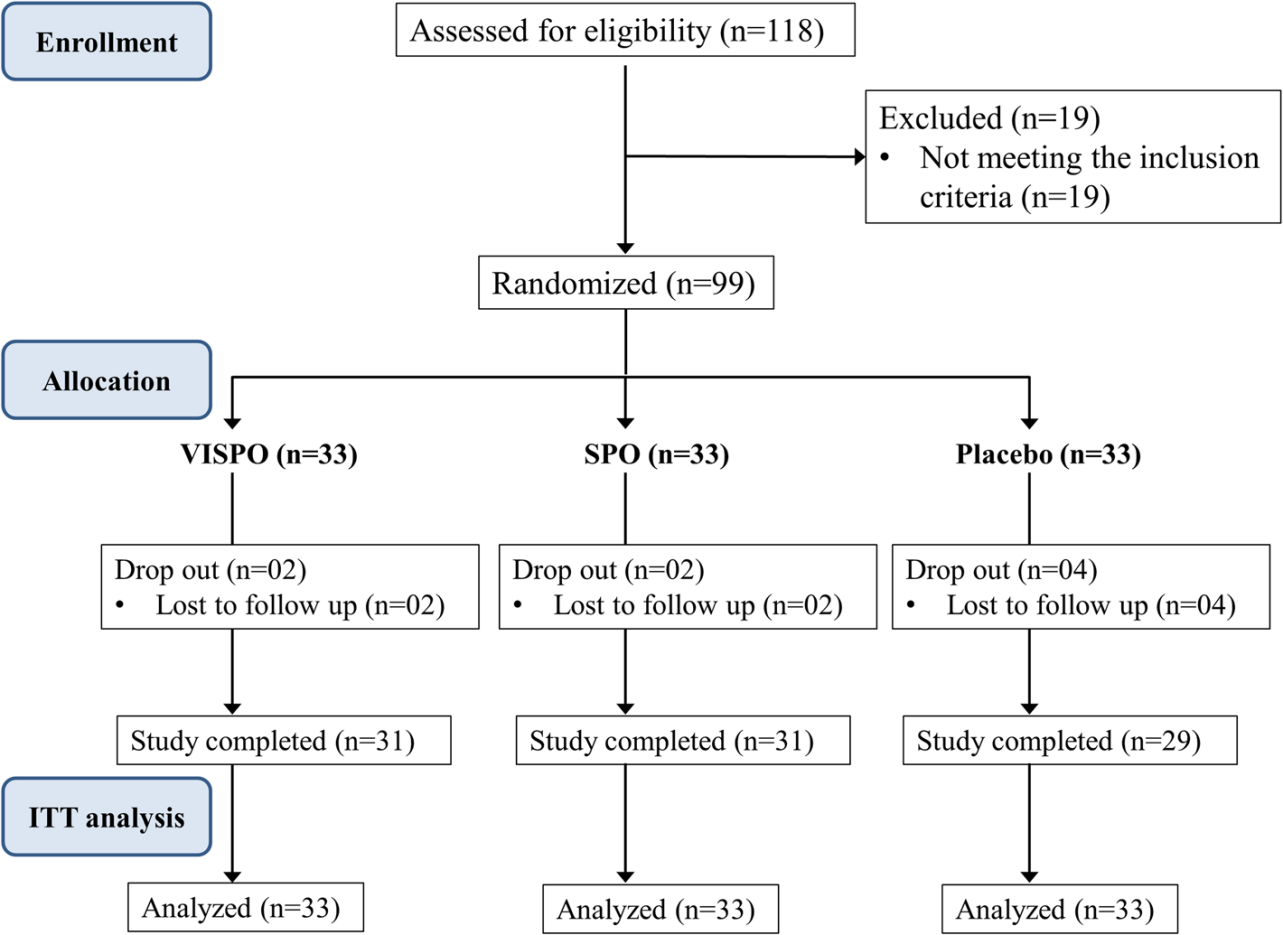
Subject participant flow chart

A total of 118 human volunteers were screened of which 99 subjects meeting the inclusion criteria were enrolled in the study. Of the 99 subjects randomized to three treatment groups (*n* = 33 in each group), 91 subjects completed the study. There were 8 dropouts in the study (reason: lost to follow up). The intention-to-treat (ITT) analysis was used to assess the outcome of the study. The participant flow through the study is presented in Fig. 2. The demographic characteristics of subjects between the groups were not significantly different (Table 2). The efficacy and safety analysis were performed using ITT population.

**Table 2:** Demographic characteristics of subjects

Variable	Intervention (VISPO) *(n* = 33)	Comparator (SPO) (*n* = 33)	Placebo (*n* = 33)	*p*-value*
Age (Years)	57.76 ± 7.25	57.48 ± 7.04	55.18 ± 8.56	0.327
Weight (kg)	61.09 ± 5.80	58.99 ± 4.89	60.87 ± 5.18	0.213
Height (cm)	158.94 ± 3.63	157.82 ± 4.16	157.82 ± 4.55	0.448
BMI (kg/m^2^)	24.18 ± 2.12	23.69 ± 1.78	24.46 ± 2.10	0.290

*N* No of subjects; *BMI* Body Mass Index; Date presented as: Mean ± Standard deviation

*p values were derived from ANOVA

The comparative efficacy of VISPO against the conventional saw palmetto oil was evaluated primarily using IPSS, ADAM and AMS scores assessed at visit 1 (baseline), visit 2 (6 weeks) and visit 3 (12 weeks) of the study. At baseline there was no significant difference in the IPSS score between the groups while the values were decreased in VISPO and SPO groups during the study (Table 3). There was a significant decrease in the IPSS score from baseline value of 20.00 ± 4.41 to 18.06 ± 4.14 at visit 2 (*p* < 0.001) and 16.82 ± 4.03 at visit 3 (*p* < 0.001). The values were not significant for SPO group. In the placebo group there was a slight increase in IPSS score during the intervention period. The change in IPSS value from baseline to the end of treatment (visit 3) was significant in saw palmetto oil treated groups as compared to placebo (*p* < 0.001).

**Table 3:** Summary of International Prostate Symptom Score (IPSS) by visit and treatment

Visit	Observed value				Change from baseline						
	Intervention (VISPO) *n* = 33	Comparator (SPO) *n* = 33	Placebo *n* = 33	*p*-value**	Intervention (VISPO) *n* = 33	*p*-value*	Comparator (SPO) *n* = 33	*p*-value*	Placebo *n* = 33	*p*-value*	*p*-value**
Visit 1	20.00 ± 4.41	19.45 ± 3.82	20.00 ± 3.74	0.815^(1)^							
Visit 2	18.06 ± 4.14^a^	19.09 ± 3.89^ab^	20.70 ± 3.37^b^	0.021^(1)^	−1.94 ± 1.85^a^	< 0.001	−0.36 ± 1.98^b^	0.300	0.70 ± 1.38^b^	0.007	< 0.001^(1)^
Visit 3	16.82 ± 4.03^a^	18.55 ± 4.22^ab^	20.73 ± 4.87^b^	0.002^(1)^	−3.18 ± 3.14^a^	< 0.001	−0.91 ± 3.34^ab^	0.128	0.73 ± 5.01^b^	0.410	< 0.001^(1)^

Data are presented as Mean ± SD; *n* = No of subjects

^a-b^ values within a row with different superscript letters differ (*p < 0.05*), as analysed by one-way ANOVA

*p values were compared within each group from baseline using paired t test

**p values were compared between groups

^1)^*P* values were derived from ANOVA, and Scheffe test was used to post hoc test

In the present study ADAM questionnaire was used to screen for the severity of androgen deficiency in men (Table 4). The ITT population analysis revealed that VISPO group subjects showed a significant reduction of ADAM score from baseline (4.33 ± 1.67) to the end of study (3.73 ± 1.74) (*p* < 0.001) while SPO and placebo groups showed an increase in the mean ADAM score. The change in the score from baseline to visit 3 of VISPO group (− 0.61 ± 1.06) was significant (*p* < 0.01) compared to placebo (0.15 ± 0.94).

**Table 4:** Summary of Androgen deficiency in the aging male (ADAM) scoring by visit and treatment

Visit	Observed value				Change from baseline						
	Intervention (VISPO) *n* = 33	Comparator (SPO) *n* = 33	Placebo *n* = 33	*p*-value**	Intervention (VISPO) *n* = 33	*p*-value*	Comparator (SPO) *n* = 33	*p*-value*	Placebo *n* = 33	*p*-value*	*p*-value**
Visit 1	4.33 ± 1.67	4.15 ± 1.68	4.30 ± 1.76	0.898^(1)^							
Visit 2	3.97 ± 1.74	4.12 ± 1.69	4.48 ± 1.73	0.461^(1)^	−0.36 ± 0.70^a^	0.005	−0.03 ± 0.53^ab^	0.744	0.18 ± 0.68^b^	0.136	0.007^(2)^
Visit 3	3.73 ± 1.74	4.21 ± 1.43	4.45 ± 1.64	0.179^(1)^	−0.61 ± 1.06^a^	0.002	0.06 ± 0.90^b^	0.701	0.15 ± 0.94^b^	0.361	0.003^(1)^

Data are presented as Mean ± SD; *n* = No of subjects

^a-b^ values within a row with different superscript letters differ (*p < 0.05*), as analysed by one-way ANOVA

*p values were compared within each group from baseline using paired t test

**p values were compared between groups

^1)^*P* values were derived from ANOVA, and Scheffe test was used to post hoc test

^2)^*P* values were derived from Kruskal-Wallis test, and Dunnett T3 test was used to post hoc test

Further AMS scale was used to assess the symptoms of ageing and to measure the changes in severity of symptoms pre- and post-intervention (Table 5). AMS scale has three dimensions (sub-scales) i.e., psychological, somatic, and sexual subscale. The composite scores for each of the three sub-scales is based on adding up the scores of the items of the respective dimensions. The AMS total score from baseline to end of treatment decreased considerably in VISPO group (*p* < 0.001). SPO group showed non-significant reduction in the mean score. The change in total AMS score from baseline to visit 3 of VISPO (− 3.64 ± 4.76) and SPO (− 1.12 ± 4.14) groups were significant (*p* < 0.001) when compared to placebo (1.70 ± 3.37). Similar trend was observed in the AMS subscale analyses (Table 6). Importantly, there was a significant reduction (*p* < 0.001) in the AMS sexual subscale from baseline to the end of treatment in VISPO (− 1.24 ± 1.84) and SPO (− 0.12 ± 1.47) groups as compared to placebo (0.73 ± 1.26).

**Table 5:** Summary of total Aging male symptoms (AMS) score by visit and treatment

Visit	Observed value				Change from baseline						
	Intervention (VISPO) *n* = 33	Comparator (SPO) *n* = 33	Placebo *n* = 33	*p*-value**	Intervention (VISPO) *n* = 33	*p*-value*	Comparator (SPO) *n* = 33	*p*-value*	Placebo *n* = 33	*p*-value*	*p*-value**
Visit 1	45.06 ± 9.28	44.64 ± 9.05	45.42 ± 10.39	0.946^1)^							
Visit 2	43.52 ± 8.61	43.97 ± 9.01	46.06 ± 9.66	0.483^1)^	−1.55 ± 2.18^a^	< 0.001	− 0.67 ± 1.80^a^	0.041	0.64 ± 1.71^b^	0.040	< 0.001^2)^
Visit 3	41.42 ± 7.52^a^	43.52 ± 8.76^ab^	47.12 ± 9.73^b^	0.031^1)^	− 3.64 ± 4.76^a^	< 0.001	−1.12 ± 4.14^a^	0.129	1.70 ± 3.37^b^	0.007	< 0.001^2)^

Data are presented as Mean ± SD; *n* = No of subjects

^a-b^ values within a row with different superscript letters differ (*p < 0.05*), as analysed by one-way ANOVA

*p values were compared within each group from baseline using paired t test

**p values were compared between groups

^1)^*P* values were derived from ANOVA, and Scheffe test was used to post hoc test

^2)^*P* values were derived from Kruskal-Wallis test, and Dunnett T3 test was used to post hoc test

**Table 6:** Summary of Aging male symptoms (AMS) subscale analysis

**AMS psychological score**											
Visit	**Observed value**				**Change from baseline**						
	Intervention (VISPO) *n* = 33	Comparator (SPO) *n* = 33	Placebo *n* = 33	*p*-value**	Intervention (VISPO) *n* = 33	*p*-value*	Comparator (SPO) *n* = 33	*p*-value*	Placebo *n* = 33	*p*-value*	*p*-value**
Visit 1	12.64 ± 4.08	12.97 ± 3.58	13.79 ± 4.92	0.559^2)^							
Visit 2	12.58 ± 3.75	12.82 ± 3.40	13.79 ± 4.72	0.510^2)^	− 0.06 ± 0.75	0.645	− 0.15 ± 0.83	0.304	0.00 ± 0.50	1.000	0.683^1)^
Visit 3	12.58 ± 3.67	12.73 ± 3.31	14.06 ± 4.39	0.351^2)^	− 0.06 ± 1.00	0.730	−0.24 ± 1.25	0.274	0.27 ± 1.04	0.141	0.162^1)^
**AMS somatic score**											
Visit 1	19.27 ± 3.88	19.85 ± 4.43	20.67 ± 4.26	0.403^1)^							
Visit 2	18.39 ± 3.64	19.45 ± 4.37^ab^	20.85 ± 3.97^b^	0.049^1)^	−0.88 ± 1.14^a^	< 0.001	− 0.39 ± 0.90^a^	0.017	0.18 ± 0.58^b^	.083	< 0.001^2)^
Visit 3	16.97 ± 3.10^a^	19.12 ± 4.52^ab^	20.79 ± 3.90^b^	0.001^2)^	−2.30 ± 2.65^a^	< 0.001	−0.73 ± 2.30^b^	0.078	0.12 ± 2.06^b^	.737	< 0.001^1)^
**AMS sexual score**											
Visit 1	13.15 ± 3.28^a^	11.82 ± 2.48^ab^	10.97 ± 2.89^b^	0.011^1)^							
Visit 2	12.55 ± 3.10	11.70 ± 2.64	11.42 ± 2.69	0.247^1)^	−0.61 ± 0.97^a^	0.001	−0.12 ± 0.82^ab^	0.402	0.45 ± 1.06^b^	.020	< 0.001^1)^
Visit 3	11.91 ± 2.96	11.70 ± 2.48	11.70 ± 2.65	0.935^1)^	−1.24 ± 1.84^a^	<.001	−0.12 ± 1.47^b^	0.640	0.73 ± 1.26^C^	.002	< 0.001^2)^

Data are presented as Mean ± SD; *n* = No of subjects

^a-b^ values within a row with different superscript letters differ (*p < 0.05*), as analysed by one-way ANOVA

*p values were compared within each group from baseline using paired t test

**p values were compared between groups

^1)^*P* values were derived from ANOVA, and Scheffe test was used to post hoc test

^2)^*P* values were derived from Kruskal-Wallis test, and Dunnett T3 test was used to post hoc test

Table 7 shows the urodynamic measurements obtained before and after the intervention period. The residual urine volume decreased significantly (*p* < 0.001) in VISPO group (from 112.55 ± 36.19 mL to 99.79 ± 29.27 mL) after the 12-week treatment. SPO group showed a slight improvement in residual urine volume, though not to a significant extent (110.12 ± 34.71 mL to 108.24 ± 33.22 mL). A significant improvement (*p* < 0.001) of Qmax was observed in the VISPO group (from 11.85 ± 2.06 mL/s to 14.27 ± 2.54 mL/s). In the SPO group a non-significant increase in the Qmax was recorded from 12.33 ± 1.61 mL/s to 12.82 ± 1.72 mL/s.

**Table 7:** Summary of urodynamic measurements by visit and treatment

**Residual urine volume (mL)**											
Visit	Observed value				Change from baseline						
	Intervention (VISPO) *n* = 33	Comparator (SPO) *n* = 33	Placebo *n* = 33	*p*-value**	Intervention (VISPO) *n* = 33	*p*-value*	Comparator (SPO) *n* = 33	*p*-value*	Placebo *n* = 33	*p*-value*	*p*-value**
Visit 1	112.55 ± 36.19	110.12 ± 34.71	114.17 ± 30.67	0.888^(1)^	−12.76 ± 14.44^a^	< 0.001	−1.88 ± 10.93^b^	0.331	1.11 ± 13.89^b^	0.649	< 0.001^(1)^
Visit 3	99.79 ± 29.27	108.24 ± 33.22	115.28 ± 33.25	0.149^(1)^							
**Qmax (mL/s)**											
Visit	Observed value				Change from baseline						
	Intervention (VISPO) *n* = 33	Comparator (SPO) *n* = 33	Placebo *n* = 33	*p*-value**	Intervention (VISPO) *n* = 33	*p*-value*	Comparator (SPO) *n* = 33	*p*-value*	Placebo *n* = 33	*p*-value*	*p*-value**
Visit 1	11.85 ± 2.06	12.33 ± 1.61	12.27 ± 1.92	0.522^(1)^	2.42 ± 2.61^a^	< 0.001	0.48 ± 1.56^b^	0.084	−0.64 ± 2.28^b^	0.118	< 0.001^(1)^
Visit 3	14.27 ± 2.54^a^	12.82 ± 1.72^b^	11.64 ± 1.41^c^	< 0.001^(2)^							

Data are presented as Mean ± SD; *n* = No of subjects

^a-b^ values within a row with different superscript letters differ (*p < 0.05*), as analysed by one-way ANOVA

*p values were compared within each group from baseline using paired t test

**p values were compared between groups

^1)^*P* values were derived from ANOVA, and Scheffe test was used to post hoc test

^2)^*P* values were derived from Kruskal-Wallis test, and Dunnett T3 test was used to post hoc test

Assessment of serum BPH markers are presented in Table 8. Serum levels of PSA was decreased in the VISPO group from 2.77 ± 0.56 ng/mL to 2.70 ± 0.59 ng/mL while it was increased in SPO and placebo groups after the treatment. The subjects in VISPO group showed an increase in free testosterone levels (6.23 ± 1.16 to 6.46 ± 1.43) after treatment (*p* < 0.05). On the contrary there was a reduction of serum levels of free testosterone in SPO and placebo groups. The change in free testosterone from baseline to end of treatment was significant in VISPO group as compared to placebo (*p* < 0.05). There was a reduction in total testosterone noted in all the treatment groups. However, the data were not significant. 5α-reductase activity was non-significantly reduced in VISPO group from baseline to the end of 12-week treatment (445.90 ± 105.40 ng/L to 438.24 ± 98.56). However, it was noticed that unexpectedly, the enzyme activity was increased in SPO group towards the end of study.

**Table 8:** Summary of BPH marker analysis

Variable	Observed value				Change from baseline						
	Intervention (VISPO) *n* = 33	Comparator (SPO) *n* = 33	Placebo *n* = 33	*p*-value**	Intervention (VISPO) *n* = 33	*p*-value*	Comparator (SPO) *n* = 33	*p*-value*	Placebo *n* = 33	*p*-value*	*p*-value**
**Prostate specific antigen (PSA) (ng/ml)**											
Visit 1	2.77 ± 0.56	2.80 ± 0.72	2.83 ± 0.52	0.931^(1)^							
Visit 3	2.70 ± 0.59	2.99 ± 0.66	3.01 ± 0.54	0.072^(1)^	−0.07 ± 0.032^a^	0.233	0.19 ± 0.40^b^	0.011	0.18 ± 0.40^b^	0.013	0.008^(1)^
**5α-reductase (ng/L)**											
Visit 1	445.90 ± 105.40	434.32 ± 93.76	441.31 ± 96.32	0.891^(1)^							
Visit 3	438.24 ± 98.56	440.40 ± 101.24	444.08 ± 138.16	0.978^(1)^	−7.66 ± 60.60	0.473	6.08 ± 51.46	0.502	2.77 ± 95.49	0.868	0.720^(1)^
**Total testosterone (ng/L)**											
Visit 1	459.62 ± 131.80	490.54 ± 166.66	433.50 ± 138.80	0.290^(1)^							
Visit 3	454.86 ± 137.31	481.02 ± 151.57	408.44 ± 140.43	0.119^(1)^	−4.76 ± 56.96	0.634	−9.52 ± 52.69	0.307	−25.07 ± 37.86	0.001	0.229^(1)^
**Free testosterone (ng/dL)**											
Visit 1	6.23 ± 1.16	6.26 ± 1.73	5.83 ± 1.31	0.394^(1)^							
Visit 3	6.46 ± 1.43	6.15 ± 1.62	5.64 ± 1.30	0.075^(1)^	0.23 ± 0.77^a^	0.093	−0.11 ± 0.60^ab^	0.294	−0.19 ± 0.43^b^	0.015	0.015^(1)^

Data presented as: Mean ± SD; *N* = No of subjects; ^a-c^ values within a row with different superscript letters differ (*p < 0.05*), as analyzed by one-way ANOVA

*p values were compared within each group from baseline using paired t test;

**p values were compared between groups

^1)^*P* values were derived from ANOVA, and Scheffe test was used to post hoc test

^2)^*P* values were derived from Kruskal-Wallis test, and Dunnett T3 test was used to post hoc test

**Fig. 3:**
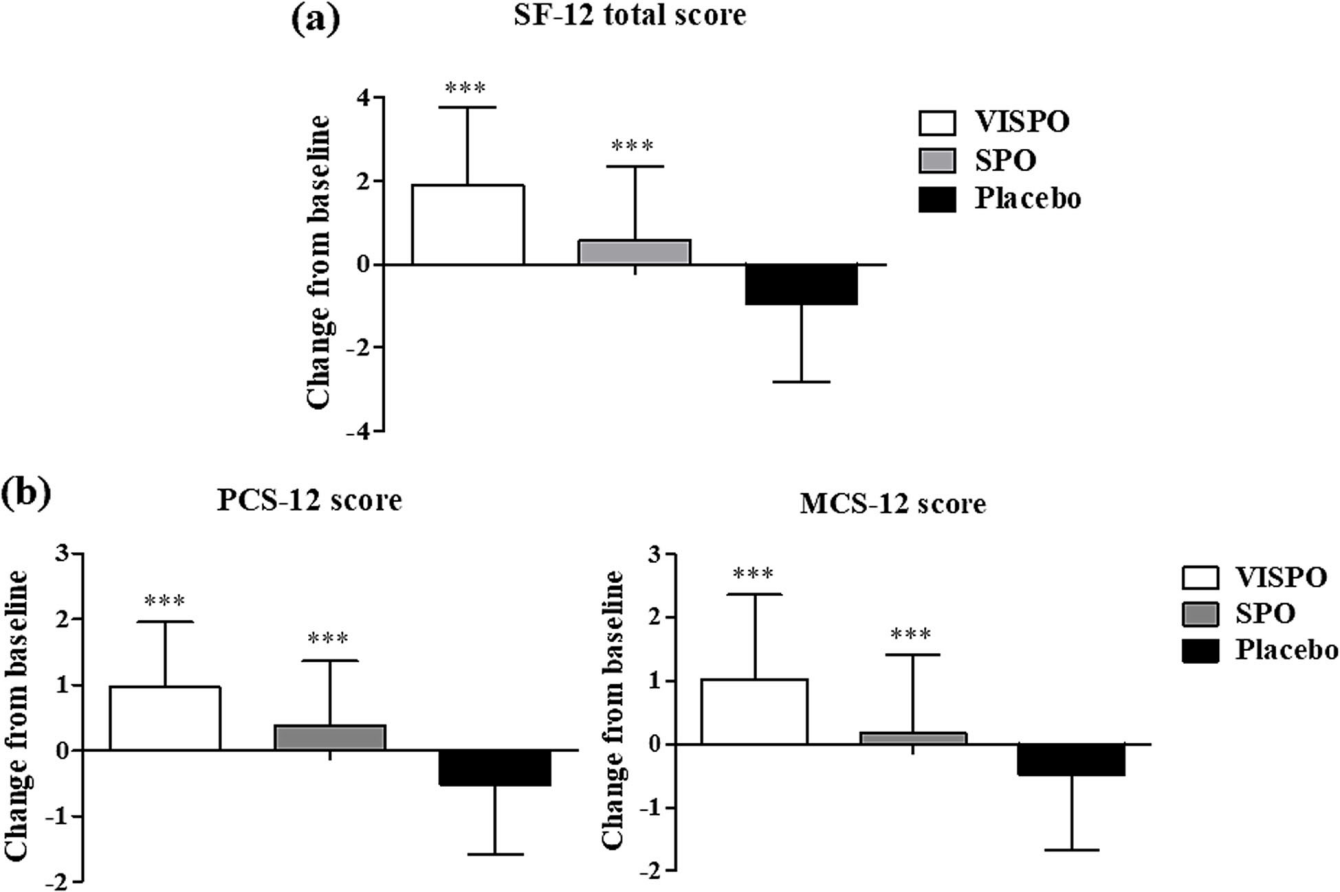
Mean change in SF-12 score of the study participants. The changes from baseline (visit 1) to the end of treatment (visit 3) in SF-12 total score (**a**), physical health composite (PCS-12) score and mental health composite (MCS-12) score (**b**) . The values are represented as mean ± SD (*n* = 33 in each group). The data were analysed by one way ANOVA followed by Scheffe test. ***p < 0.001 vs. placebo

The Short-Form Health Survey (SF-12) is one of the most widely used questionnaire for assessing self-reported health-related quality of life. VISPO group subjects demonstrated a significant improvement (*p* < 0.001) in the quality of life as evident from the increase in the mean total score. Comparatively, SPO group showed moderate increase in the SF-12 total score. Both VISPO and SPO groups showed significant change in mean score from visit 1 to visit 3 as compared to placebo. The subset analysis revealed that VISPO group also showed significant improvement in PCS-12 and MCS-12 scores after 12-week treatment (*p* < 0.001), Fig. 3 shows the change in SF-12 scores from baseline to the end of treatment.

No serious adverse events (SAEs) were recorded during the study. The investigational products were well tolerated among the subjects. There were no significant alterations in the biochemical and haematological parameters among the subjects during the intervention period. The results of safety assessment are provided in **supplementary file** **4**.

## Discussion

SP extracts used extensively for treatment of LUTS in traditional medicine have been clinically validated for improving the conditions such as BPH [12]. These medicinal attributes of SP extracts are due to the synergistic effect of phytosterols and fatty acids [13]. Previously, we have demonstrated the improved efficacy of SP oil containing 3% β-sitosterol (VISPO) compared to the conventional oil in alleviating the symptoms of BPH in rats [11]. Results from preclinical studies prompted us to conduct the clinical trial to further confirm the effectiveness of VISPO.

Here we have clinically evaluated the efficacy of VISPO in comparison with conventional SP oil (SPO). Considering the prevalence of mild to moderate BPH symptoms in men from the age of 40 to 60 and above [14–16], subjects aged 40–65 years were included in the trial. This study was primarily focused on the changes in BPH symptoms assessed subjectively using the validated questionnaires. IPSS score is a convenient tool for the assessment of BPH-specific symptoms in order to evaluate the treatment outcomes [17, 18]. There was a significant decrease in the IPSS score from baseline (visit 1) to the end of treatment in VISPO and SPO groups indicating amelioration of BPH symptoms. Further we have used AMS scale and ADAM score as subjective measures of androgen deficiency. The age-related androgen deficiency associated with BPH must be considered for an effective therapeutic strategy [19]. The mean scores were markedly reduced in VISPO group following the treatment. It was interesting to note that the AMS sexual score was significantly reduced in VISPO group as compared to placebo. Several treatment strategies and surgical therapies for BPH have been associated with sexual side effects [20]. It is an important observation from our study that a 12-week ingestion of β-sitosterol enriched SP oil could markedly improve the sexual function among the subjects while the conventional SP oil exerted the moderate efficacy. In accordance with the subjective measurements, the urodynamic parameters such as PVR and Qmax were significantly improved in VISPO group as compared to placebo. The mean changes from baseline to the end of treatment were appreciable in VISPO group than SPO group. Previously, Oki et al., have demonstrated that SP extract could significantly improve the urodynamic symptoms and micturition reflex in rats [21].

One of the important age-related symptoms is the testosterone deficiency which may subsequently lead to the adverse effects on androgen-responsive organ functions [22, 23]. Longitudinal studies have found dramatic reduction in free testosterone levels in aging men [24, 25]. In the present study, treatment with VISPO showed slight increase in the free testosterone and was significantly better as compared to SPO treated group.

Interestingly, there was an insignificant reduction in the 5α-reductase activity in VISPO group from baseline to the end of treatment. Our findings suggest that VISPO could exert its efficacy through other mechanisms such as alpha-adrenergic or muscarinic receptor blockage. Previously, SP extract was shown to reduce the bladder muscarinic and purinergic receptors and micturition frequency in a rat model of cystitis [26]. Suzuki et al., reported significant binding activity of SP extract on autonomic receptors in rats [27]. Studies from Aebi et al. reported oleic acid, lauric acid, myristic acid and linoleic acid as major fatty acids in SP extract having potential binding activity with alpha-adrenergic or muscarinic receptors in addition to 5α-reductase inhibitory activity [9, 28]. In accordance with these reports and the results of present study, a synergistic effect of fatty acids and β-sitosterol could contribute to the improved functional attributes of VISPO possibly with multiple mode of action. Other explanatory mechanisms of action include the anti-inflammatory and apoptotic effects [29]. In a multicentered pilot study, Vela Navarrete et al.*,* reported that a 3-month treatment with 160 mg SP extract (Permixon®) could significantly reduce the IPSS score alongside the inflammatory parameters IL-1β and TNF-α in prostate tissues of BPH patients [30]. In another study, Permixon treatment significantly reduced the cell number and proliferative indices in BPH tissues indicating the role of SP extract in the induction of apoptosis in BPH [31]. We have previously demonstrated the anti-inflammatory and anti-proliferative effects of VISPO in preclinical model of BPH [11].

In this double-blind placebo-controlled study, we have demonstrated the improvement in BPH symptoms and androgen deficiency as a function of higher β-sitosterol content in VISPO. β-sitosterol has been previously reported to have profound influence on the prostate health [32]. Berges et al. have published their research findings on β-sitosterol wherein a 6-month administration of 20 mg of the compound significantly reduced the prostate size, increased the urine flow rate, and improved the mean voiding time and quality of life score in BPH patients [33]. In another multicentric, placebo-controlled, double-blind study daily intake of β-sitosterol markedly reduced the IPSS score with an increased Qmax and decreased post-void residual urine volume among BPH patients [34]. β-sitosterol was previously shown to inhibit the 5α-reductase activity in hamster prostate [35]. Our results are in line with these literature as ingestion of 400 mg/day VISPO, the SP extract with higher β-sitosterol content showed superior efficacy compared to conventional SP oil. The overall increment in efficacy parameters observed in VISPO group could be due to the synergistic effect of higher β-sitosterol content and the conventionally present fatty acids in saw palmetto extract. Collectively, the different constituents in the extract may contribute to the efficacy of the extract via multiple mode of action [36].

## Conclusion

The present study provides first ever clinical evidence on the improved efficacy of saw palmetto oil due to the enriched content of β-sitosterol. This comparative study clearly recommends the use of a phytosterol-enriched saw palmetto oil as a functional ingredient in dietary supplements for effective management of symptomatic BPH.

## Supplementary information

**Additional file 1: Supplementary file 1.** Schedule of study procedures

**Additional file 2: Supplementary file 2.** Sample size calculation

**Additional file 3: Supplementary file 3.** Questionnaires used in the study (Subjective assessment)

**Additional file 4: Supplementary file 4.** Safety evaluation data

## Data Availability

The data sets used and/or analyzed during the current study available from the corresponding author on reasonable request.

## Abbreviations

BPH: Benign prostate hyperplasia
SF-12: The 12-item short form survey
PSA: Prostate specific antigen
SP: Saw palmetto
IPSS: International prostate symptom score
AMS scale: Aging males’ symptoms scale
ADAM: Androgen deficiency in aging males
LUTS: Lower urinary tract symptoms
PCS-12: Physical health composite score
MCS-12: Mental health composite score
ITT: Intention-to-treat
ANOVA: Analysis of variance
AST: Aspartate aminotransferase
ALT: Alanine aminotransferase
BUN: Blood urea nitrogen
Qmax: Maximum urine flowrate
5ARIs: 5α-Reductase inhibitors
