# Supplementary material for: A double blind, placebo-controlled randomized comparative study on the efficacy of phytosterol-enriched and conventional saw palmetto oil in mitigating benign prostate hyperplasia and androgen deficiency
Source: BMC Urol. 2020 Jul 3;20:86. doi: 10.1186/s12894-020-00648-9 (PMC7333342; doi:10.1186/s12894-020-00648-9)
Supplement: Supplementary file 1 — Additional file 1: Supplementary file 1. Schedule of study procedures [file 12894_2020_648_MOESM1_ESM.docx]

**Schedule of study procedures**

| Phase | Screening/  Baseline | Study Visits | |
| --- | --- | --- | --- |
| Visit | Visit 1 | Visit 2 | Visit 3 |
| Day | Day 1 | Day 42 ± 3 | Day 84 ± 3 |
| Informed consent | X |  |  |
| Inclusion and exclusion criteria assessment | X |  |  |
| Demography | X |  |  |
| Vital signs (SBP, DBP, HR, Temp.) | X | X | X |
| Physical examination | X | X | X |
| International Prostatic Symptom Score | X | X | X |
| Aging Male Symptoms Scale | X | X | X |
| Androgen Deficiency in the Aging Male | X | X | X |
| Medical history | X | X | X |
| Prior medication history | X |  |  |
| Concomitant medication history |  | X | X |
| Randomization | X |  |  |
| Uroflowmetry | X |  | X |
| Ultrasonography: PVR measurement | X |  | X |
| Laboratory assessment* | X |  | X |
| Short-Form Health Survey (SF-12) questionnaire | X | X | X |
| Dispense of Investigational Products | X | X |  |
| Issue of subject diary | X | X |  |
| Safety assessment |  | X | X |
| Collection and review of subject diary |  | X | X |
| IP Accountability and Compliance |  | X | X |

SBP: Systolic Blood Pressure, DBP: Diastolic Blood Pressure, HR: Heart Rate; Temp.: Temperature; PVR: Postvoid Residual Urine Volume

*Laboratory parameters: Prostate specific antigen, Serum creatinine, Blood urea nitrogen, Complete blood count, Aspartate aminotransferase, Alanine aminotransferase, Urine analysis, 17-beta-Hydroxysteroid dehydrogenase, 3-beta–Hydroxysteroid dehydrogenase, 5 alpha-reductase, Testosterone and Free testosterone
