# Supplementary material for: A double blind, placebo-controlled randomized comparative study on the efficacy of phytosterol-enriched and conventional saw palmetto oil in mitigating benign prostate hyperplasia and androgen deficiency
Source: BMC Urol. 2020 Jul 3;20:86. doi: 10.1186/s12894-020-00648-9 (PMC7333342; doi:10.1186/s12894-020-00648-9)
Supplement: Supplementary file 2 — Additional file 2: Supplementary file 2. Sample size calculation [file 12894_2020_648_MOESM2_ESM.pdf]

**Power and Sample Size Determination**

Assuming the mean difference for all groups 2.35 between **VISPO, SPO** and **placebo and** standard deviation 2.45, and approximately 33 subjects randomized to each treatments, this study will have 80% power at the level of 0.05 (2-sided) using a **One-way ANOVA between groups**. This is considered for comparing three treatment groups on a measurement outcome variable through the ANOVA. To allow for an estimated 12% potential dropout rate, this study plans to enroll approximately 99 subjects to ensure that at least 87 to complete. The assumed effect size is based on differences measured between **VISPO, SPO** and **placebo** in previous studies conducted with similar drug formulations in male subjects.

```
ss.1way(k=3, alpha=0.05, beta=0.1, delta=2.35, sigma=2.45, B=100)
```

```
     Balanced one-way analysis of variance sample size adjustment

              k = 3
      sig.level = 0.05
          power = 0.9
              n = 29

NOTE: n is number in each group, total sample = 87
```

A total of *N=87*, number of subjects are required in the end of the study with all the data being complete for analysis, but a proportion (*q=0.12*) are expected to drop out before the study ends. In this case, the following total number of subjects (*N1*) would have to be enrolled to ensure that the final sample size (*N*) is achieved:

$$N1 = \frac{N}{1-q} = \frac{87}{(1-0.12)} = 99$$

Where *q* is the proportion of attrition and is generally 12% in this type of studies.
