# Supplementary material for: A double blind, placebo-controlled randomized comparative study on the efficacy of phytosterol-enriched and conventional saw palmetto oil in mitigating benign prostate hyperplasia and androgen deficiency
Source: BMC Urol. 2020 Jul 3;20:86. doi: 10.1186/s12894-020-00648-9 (PMC7333342; doi:10.1186/s12894-020-00648-9)
Supplement: Supplementary file 3 — Additional file 3: Supplementary file 3. Questionnaires used in the study (Subjective assessment) [file 12894_2020_648_MOESM3_ESM.pdf]

International Prostate Symptom Score (IPSS) Scoring

	Not at all	Less than 1 time in 5	Less than half the time	About half the time	More than half the time	Almost always	**Your score**
**1. Incomplete emptying**<br>Over the past month, how often have you had a sensation of not emptying your bladder completely after you finish urinating?	0	1	2	3	4	5	
**2. Frequency**<br>Over the past month, how often have you had to urinate again less than two hours after you finished urinating?	0	1	2	3	4	5	
**3. Intermittency**<br>Over the past month, how often have you found you stopped and started again several times when you urinated?	0	1	2	3	4	5	
**4. Urgency**<br>Over the last month, how difficult have you found it to postpone urination?	0	1	2	3	4	5	
**5. Weak stream**<br>Over the past month, how often have you had a weak urinary stream?	0	1	2	3	4	5	
**6. Straining**<br>Over the past month, how often have you had to push or strain to begin urination?	0	1	2	3	4	5	

	None	1 time	2 times	3 times	4 times	5 times or more	**Your score**
**7. Nocturia**<br>Over the past month, many times did you most typically get up to urinate from the time you went to bed until the time you got up in the morning?	0	1	2	3	4	5	

Total IPSS score	

Total score: 0-7 Mildly symptomatic; 8-19 moderately symptomatic; 20-35 severely symptomatic.

**Quality of life due to urinary symptoms**	Delighted	Pleased	Mostly satisfied	Mixed – about equally satisfied and dissatisfied	Mostly dissatisfied	Unhappy	Terrible
If you were to spend the rest of your life with your urinary condition the way it is now, how would you feel about that?	0	1	2	3	4	5	6

Aging Male Symptoms Scale (AMS)

Which of the following symptoms apply to you at this time? Please, mark the appropriate box for each symptom. For symptoms that do not apply, please mark “none”.

Symptom		None	Mild	Moderate	Severe	Extremely severe
	Score =	1	2	3	4	5
**1. Decline in your feeling of general well-being** (general state of health, subjective feeling)		☐	☐	☐	☐	☐
**2. Joint pain and muscular ache** (lower backpain, joint pain, pain in a limb, general back ache)		☐	☐	☐	☐	☐
**3. Excessive sweating** (unexpected/sudden episodes of sweating, hot flushes independent of strain)		☐	☐	☐	☐	☐
**4. Sleep problems** (difficulty in falling asleep, difficulty in sleeping through, waking up early and feeling tired, poor sleep, sleeplessness.		☐	☐	☐	☐	☐
**5. Increased need for sleep, often feeling tired**		☐	☐	☐	☐	☐
**6. Irritability** (feeling aggressive, easily upset about little things, moody)		☐	☐	☐	☐	☐
**7. Nervousness** (inner tension, restlessness, feeling fidgety)		☐	☐	☐	☐	☐
**8. Anxiety** (Feeling panicky)		☐	☐	☐	☐	☐
**9. Physical exhaustion/lacking vitality** (general decrease in performance, reduced activity, lacking interest in leisure activities, feeling of getting less done, of achieving less, of having to force oneself to undertake activities)		☐	☐	☐	☐	☐
**10. Decrease in muscular strength** (feeling of weakness)		☐	☐	☐	☐	☐
**11. Depressive mood** (feeling down, sad, on the verge of tears, lack of drive, moodswings, feeling nothing is of any use)		☐	☐	☐	☐	☐
**12. Feeling that you have passed your peak**		☐	☐	☐	☐	☐
**13. Feeling burntout, having hit rock- bottom**		☐	☐	☐	☐	☐
**14. Decrease in beard growth**		☐	☐	☐	☐	☐
**15. Decrease in ability/frequency to perform sexually**		☐	☐	☐	☐	☐
**16. Decrease in the number of morning erections**		☐	☐	☐	☐	☐
**17. Decrease in sexual desire/libido** (lacking pleasure in sex, lacking desire for sexual intercourse)		☐	☐	☐	☐	☐
**Have you got any other major symptoms?**	Yes	☐	No	☐		

If yes, please describe:

Thank you very much for your cooperation

Androgen Deficiency in the Aging Male (ADAM) Questionnaire

Please answer the following questions honestly

		Yes	No
1.	Do you have a decrease in libido (sex drive)?	☐	☐
2.	Do you have a lack of energy?	☐	☐
3.	Do you have a decrease in strength and/or endurance?	☐	☐
4.	Have you lost height?	☐	☐
5.	Have you noticed a decreased "enjoyment of life"?	☐	☐
6.	Are you sad and/or grumpy?	☐	☐
7.	Are your erections less strong?	☐	☐
8.	Have you noticed a recent deterioration in your ability to play sports?	☐	☐
9.	Are you falling asleep after dinner?	☐	☐
10.	Has there been a recent deterioration in your work performance?	☐	☐

Short-Form Health Survey (SF-12) Questionnaire

This information will help your doctors keep track of how you feel and how well you are able to do your usual activities. Answer every question by placing a check mark on the line in front of the appropriate answer.

Mark the choice that best applies to you and your situation with an "X" in the box.

1. In general, would you say your health is:

- ☐ Excellent (1)
- ☐ Very Good (2)
- ☐ Good (3)
- ☐ Fair (4)
- ☐ Poor (5)

The following two questions are about activities you might do during a typical day. Does YOUR HEALTH NOW LIMIT YOU in these activities? If so, how much?

2. MODERATE ACTIVITIES, such as moving a table, pushing a vacuum cleaner, bowling, or playing golf:

- ☐ Yes, Limited A Lot (1)
- ☐ Yes, Limited A Little (2)
- ☐ No, Not Limited At All (3)

3. Climbing SEVERAL flights of stairs:

- ☐ Yes, Limited A Lot (1)
- ☐ Yes, Limited A Little (2)
- ☐ No, Not Limited At All (3)

During the PAST 6 WEEKS have you had any of the following problems with your work or other regular activities AS A RESULT OF YOUR PHYSICAL HEALTH?

4. ACCOMPLISHED LESS than you would like:

- ☐ Yes (1)
- ☐ No (2)

5. Were limited in the KIND of work or other activities:

- ☐ Yes (1)
- ☐ No (2)

During the PAST 6 WEEKS, were you limited in the kind of work you do or other regular activities AS A RESULT OF ANY EMOTIONAL PROBLEMS (such as feeling depressed or anxious)?

6. ACCOMPLISHED LESS than you would like:

- ☐ Yes (1)
- ☐ No (2)

7. Didn't do work or other activities as CAREFULLY as usual:

- ☐ Yes (1)
- ☐ No (2)

8. **During the PAST 6 WEEKS, how much did PAIN interfere with your normal work (including both work outside the home and housework)?**
   - ☐ Not At All (1)
   - ☐ A 6 Bit (2)
   - ☐ Moderately (3)
   - ☐ Quite A Bit (4)
   - ☐ Extremely (5)

The next three questions are about how you feel and how things have been DURING THE PAST 6 WEEKS. For each question, please give the one answer that comes closest to the way you have been feeling. How much of the time during the PAST 6 WEEKS?

9. **Have you felt calm and peaceful?**
   - ☐ All of the Time (1)
   - ☐ Most of the Time (2)
   - ☐ A Good Bit of the Time (3)
   - ☐ Some of the Time (4)
   - ☐ A Little of the Time (5)
   - ☐ None of the Time (6)

10. **Did you have a lot of energy?**
   - ☐ All of the Time (1)
   - ☐ Most of the Time (2)
   - ☐ A Good Bit of the Time (3)
   - ☐ Some of the Time (4)
   - ☐ A Little of the Time (5)
   - ☐ None of the Time (6)

11. **Have you felt downhearted and blue?**
   - ☐ All of the Time (1)
   - ☐ Most of the Time (2)
   - ☐ A Good Bit of the Time (3)
   - ☐ Some of the Time (4)
   - ☐ A Little of the Time (5)
   - ☐ None of the Time (6)

12. **During the PAST 6 WEEKS, how much of the time has your PHYSICAL HEALTH OR EMOTIONAL PROBLEMS interfered with your social activities (like visiting with friends, relatives, etc.)?**
   - ☐ All of the Time (1)
   - ☐ Most of the Time (2)
   - ☐ A Good Bit of the Time (3)
   - ☐ Some of the Time (4)
   - ☐ A Little of the Time (5)
   - ☐ None of the Time (6)
