# Supplementary material for: A double blind, placebo-controlled randomized comparative study on the efficacy of phytosterol-enriched and conventional saw palmetto oil in mitigating benign prostate hyperplasia and androgen deficiency
Source: BMC Urol. 2020 Jul 3;20:86. doi: 10.1186/s12894-020-00648-9 (PMC7333342; doi:10.1186/s12894-020-00648-9)
Supplement: Supplementary file 4 — Additional file 4: Supplementary file 4. Safety evaluation data [file 12894_2020_648_MOESM4_ESM.docx]

Safety evaluation

Table 1. Overall summary of adverse events (Safety population)

| Adverse Events | GROUP A (VISPO) | GROUP B (SPO) | GROUP C (Placebo) | Overall |
| --- | --- | --- | --- | --- |
|  | (N=33)  n (%) | (N=33)  n (%) | (N=33)  n (%) | (N=99)  n (%) |
| Total Number of AEs Reported | 2 (6.06) | 4 (12.12%) | 4 (12.12%) | 10 (10.10) |
| Subjects Reporting at least one AEs | 2 (6.06) | 3 (9.09) | 3 (9.09) | 8 (8.08) |
| Total Number of SAEs Reported | 0 | 0 | 0 | 0 |
| Subjects Reporting Serious AEs | 0 | 0 | 0 | 0 |
| Subjects Reporting drug-related AEs | 0 | 0 | 0 | 0 |
| Subjects Reporting AEs leading to early discontinuation | 0 | 0 | 0 | 0 |
| Number of Deaths | 0 | 0 | 0 | 0 |

Data presented as n (%). N: No of subjects (%)

Table 2. Liver function analysis (Safety population)

| Variable | Observed value | | | | Change from baseline | | | | | | |
| --- | --- | --- | --- | --- | --- | --- | --- | --- | --- | --- | --- |
|  | Intervention  (VISPO)  n=33 | Comparator  (SPO)  n=33 | Placebo  n=33 | *p*-value** | Intervention  (VISPO)  n=33 | *p*-value* | Comparator  (SPO)  n=33 | *p*-value* | Placebo  n=33 | *p*-value* | *p*-value** |
| **Aspartate aminotransferase (U/L)** | | | | | | | | | | | |
| Visit 1 | 24.40±5.68 | 23.92±5.74 | 26.39±5.51 | 0.174^(1)^ |  | | | | | | |
| Visit 3 | 24.32±6.17 | 25.04±5.51 | 26.04±6.31 | 0.508^(1)^ | -0.08±4.76 | 0.928 | 1.12±3.50 | 0.075 | -0.35±3.15 | 0.530 | 0.264^(1)^ |
| **Alanine aminotransferase (U/L)** | | | | | | | | | | | |
| Visit 1 | 27.00±5.16 | 26.79±5.59 | 28.68±5.71 | 0.312^(1)^ |  | | | | | | |
| Visit 3 | 26.62±5.60 | 27.33±5.66 | 29.04±5.58 | 0.205^(1)^ | -0.38±4.19 | 0.603 | 0.55±4.21 | 0.462 | 0.35±3.42 | 0.558 | 0.605^(1)^ |

N= No of subjects; Change: V1-V3; U/L: Units/Litre

**p* values were compared within each group from baseline using paired t test.

***p* values were compared between groups.

^1)^ P values were derived from ANOVA, and Scheffe test was used to post hoc test.

^2)^P values were derived from Kruskal-Wallis test, and Dunnett T3 test was used to post hoc test.

Table 3. Renal function analysis (Safety population)

| Variable | Observed value | | | | Change from baseline | | | | | | |
| --- | --- | --- | --- | --- | --- | --- | --- | --- | --- | --- | --- |
|  | Intervention  (VISPO)  n=33 | Comparator  (SPO)  n=33 | Placebo  n=33 | *p*-value** | Intervention  (VISPO)  n=33 | *p*-value* | Comparator  (SPO)  n=33 | *p*-value* | Placebo  n=33 | *p*-value* | *p*-value** |
| **Serum Creatinine (mg/dL)** | | | | | | | | | | | |
| Visit 1 | 1.17±0.15 | 1.18±0.17 | 1.16±0.15 | 0.879^1)^ |  | | | | | | |
| Visit 3 | 1.20±0.12 | 1.20±0.14 | 1.18±0.14 | 0.729^1)^ | 0.02±0.08 | 0.115 | 0.02±0.07 | 0.127 | 0.02±0.08 | 0.308 | 0.930^1)^ |
| **Blood Urea Nitrogen (mg/dL)** | | | | | | | | | | | |
| Visit 1 | 14.71±2.04 | 14.77±3.37 | 14.16±1.94 | 0.561^1)^ |  | | | | | | |
| Visit 3 | 14.64±1.43 | 14.77±1.22 | 14.48±1.58 | 0.713^1)^ | -0.07±2.12 | 0.847 | 0.01±3.16 | 0.993 | 0.32±2.25 | 0.414 | 0.601^1)^ |
| **Specific gravity** | | | | | | | | | | | |
| Visit 1 | 1.02±0.05 | 1.02±0.01 | 1.02±0.01 | 0.428^1)^ |  | | | | | | |
| Visit 3 | 1.01±0.01 | 1.01±0.01 | 1.02±0.01 | 0.474^1)^ | -0.01±0.05 | 0.201 | 0.00±0.01 | 0.044 | 0.00±0.01 | 0.545 | 0.376^1)^ |
| **pH** | | | | | | | | | | | |
| Visit 1 | 6.52±0.46 | 6.55±0.46 | 6.36±0.47 | 0.237^1)^ |  | | | | | | |
| Visit 3 | 6.76±0.49^a^ | 6.48±0.44^a^ | 6.70±0.47^a^ | 0.048^1)^ | 0.24±0.72 | 0.052 | -0.06±0.60 | 0.563 | 0.33±0.75 | 0.015 | 0.057^1)^ |

N= No of subjects; Change: V1-V3; U/L: Units/Litre

**p* values were compared within each group from baseline using paired t test.

***p* values were compared between groups.

^1)^ P values were derived from ANOVA, and Scheffe test was used to post hoc test.

^2)^P values were derived from Kruskal-Wallis test, and Dunnett T3 test was used to post hoc test.

Table 4. Haematology parameter analysis (Safety population)

| Variable | Observed value | | | | Change from baseline | | | | | | |
| --- | --- | --- | --- | --- | --- | --- | --- | --- | --- | --- | --- |
|  | Intervention  (VISPO)  n=33 | Comparator  (SPO)  n=33 | Placebo  n=33 | *p*-value** | Intervention  (VISPO)  n=33 | *p*-value* | Comparator  (SPO)  n=33 | *p*-value* | Placebo  n=33 | *p*-value* | *p*-value** |
| **Red Blood Cell count (million/cmm)** | | | | | | | | | | | |
| Visit 1 | 4.99±0.42 | 5.01±0.46 | 4.99±0.44 | 0.977^(1)^ |  | | | | | | |
| Visit 3 | 4.94±0.26 | 4.99±0.23 | 4.99±0.23 | 0.573^(1)^ | -0.05±0.34 | 0.425 | -0.02±0.44 | 0.623 | 0.01±0.40 | 0.939 | 0.861^(1)^ |
| **Haemoglobin (g/dL)** | | | | | | | | | | | |
| Visit 1 | 13.30±1.14 | 13.51±0.93 | 13.31±1.07 | 0.660^(1)^ |  | | | | | | |
| Visit 3 | 13.34±0.82 | 13.57±0.69 | 13.44±0.82 | 0.476^(1)^ | 0.03±0.46 | 0.680 | 0.06±0.39 | 0.401 | 0.13±0.56 | 0.194 | 0.691^(1)^ |
| **Haematocrit (%)** | | | | | | | | | | | |
| Visit 1 | 40.91±2.97 | 41.75±2.96 | 40.75±2.91 | 0.335^(1)^ |  | | | | | | |
| Visit 3 | 40.44±2.23 | 41.19±1.88 | 39.49±6.85 | 0.260^(1)^ | -0.47±2.07 | 0.205 | -0.56±2.04 | 0.123 | -1.26±6.36 | 0.264 | 0.843^(2)^ |
| **Mean Cell Volume (fL)** | | | | | | | | | | | |
| Visit 1 | 82.15±5.18 | 83.37±4.29 | 82.23±6.14 | 0.575^(1)^ |  | | | | | | |
| Visit 3 | 81.61±3.34 | 82.22±2.16 | 82.09±2.97 | 0.657^(1)^ | -0.55±4.48 | 0.487 | -1.15±4.32 | 0.135 | -0.14±6.13 | 0.897 | 0.715^(1)^ |
| **Mean Cell Haemoglobin (Pg)** | | | | | | | | | | | |
| Visit 1 | 26.73±2.18 | 27.11±2.14 | 27.49±4.40 | 0.611^(1)^ |  | | | | | | |
| Visit 3 | 26.93±1.53 | 27.04±0.88 | 26.86±1.28 | 0.844^(1)^ | 0.20±1.68 | 0.500 | -0.07±2.18 | 0.662 | -0.63±4.43 | 0.422 | 0.525^(1)^ |
| **Mean Cell Haemoglobin Concentration (%)** | | | | | | | | | | | |
| Visit 1 | 32.05±1.10 | 32.42±0.99 | 32.67±1.12 | 0.631^(1)^ |  | | | | | | |
| Visit 3 | 32.95±1.23 | 32.95±0.99 | 32.93±1.46 | 0.997^(1)^ | 0.045±1.27 | 0.051 | 0.53±1.20 | 0.017 | 0.26±1.55 | 0.342 | 0.712^(1)^ |
| **Platelet Count (cmm)** | | | | | | | | | | | |
| Visit 1 | 284030±57877 | 279091±51041 | 283788±53771 | 0.917^(1)^ |  | | | | | | |
| Visit 3 | 263752±37904 | 268745±38297 | 265227±38393 | 0.862^(1)^ | -20278±38141 | 0.005 | -10345±35830 | 0.107 | -18561±33288 | 0.003 | 0.487^(1)^ |
| **Total Leukocyte Count (cmm)** | | | | | | | | | | | |
| Visit 1 | 6566.7±1451.0 | 7218.2±1573.5 | 6975.8±1412.0 | 0.201^(1)^ |  | | | | | | |
| Visit 3 | 6910.0±1136.6 | 7344.5±1150.6 | 7331.9±1038.9 | 0.200^(1)^ | 343.3±709.9 | 0.009 | 126.3±997.4 | 0.472 | 356.2±792.8 | 0.015 | 0.559^(2)^ |

N= No of subjects; EOS: End of study; cmm: cubic millimetre; g/dl: Gram/decilitre fL: femtolitres, 10^−15^L; Pg: picograms

**p* values were compared within each group from baseline using paired t test; ***p* values were compared between groups.

^1)^ P values were derived from ANOVA, and Scheffe test was used to post hoc test.

^2)^P values were derived from Kruskal-Wallis test, and Dunnett T3 test was used to post hoc test.

Table 5. Summary of vital signs observed during the study

| Parameters | Visit | Statistics | GROUP A (VISPO) | GROUP B (SPO) | GROUP C (Placebo) | Overall |
| --- | --- | --- | --- | --- | --- | --- |
|  |  |  | (N=33) | (N=33) | (N=33) | (N=99) |
|  |  |  |  |  |  |  |
| **Temperature (°C)** | Baseline | Mean | 97.75 | 97.73 | 97.85 | 97.77 |
|  |  | SD | 0.63 | 0.61 | 0.51 | 0.58 |
|  | EOS | Mean | 97.98 | 97.87 | 97.99 | 97.94 |
|  |  | SD | 0.42 | 0.44 | 0.38 | 0.41 |
| **Pulse rate (beats/minute)** | Baseline | Mean | 79.64 | 78.45 | 80.00 | 79.36 |
|  |  | SD | 6.69 | 6.87 | 7.28 | 6.91 |
|  | EOS | Mean | 79.29 | 77.90 | 79.34 | 78.84 |
|  |  | SD | 5.99 | 5.97 | 6.10 | 5.99 |
| **Systolic Blood Pressure (mmHg)** | Baseline | Mean | 118.18 | 117.39 | 120.18 | 118.59 |
|  |  | SD | 6.29 | 7.15 | 7.18 | 6.92 |
|  | EOS | Mean | 117.55 | 117.81 | 118.76 | 118.02 |
|  |  | SD | 4.61 | 6.25 | 4.64 | 5.20 |
| **Diastolic Blood Pressure (mmHg)** | Baseline | Mean | 74.91 | 75.94 | 78.18 | 76.34 |
|  |  | SD | 6.54 | 5.84 | 6.27 | 6.31 |
|  | EOS | Mean | 74.90 | 75.16 | 77.38 | 75.78 |
|  |  | SD | 4.73 | 5.13 | 4.84 | 4.98 |

N: No of subjects; SD: Standard deviation; EOS: End of Study
